# The Place of Generic Medicines in Hospital Prescriptions: A Survey Conducted in Medical Departments of the University Hospital of Marrakesh, Morocco

**DOI:** 10.7759/cureus.62401

**Published:** 2024-06-14

**Authors:** Sanaa Zaoui, Abdellah Bouelhaz, Amal Habchane, Hafida Khalouki, Yassine Chait, Zouhour Samlani, Ayyoub Alioua, Illias Tazi

**Affiliations:** 1 Department of Pharmacology and Toxicology, Mohammed VI University Hospital, Marrakesh, MAR; 2 Bioscience and Health Laboratory, Faculty of Medicine and Pharmacy, Cadi Ayyad University, Marrakesh, MAR; 3 Department of Radiology, Mohammed VI University Hospital, Marrakesh, MAR; 4 Hepato-Gastro-Enterology, Souss Massa University Hospital, Agadir, MAR; 5 Department of Gastroenterology, Mohammed VI University Hospital, Marrakesh, MAR; 6 Science and Technology and Medical Sciences, Bioscience, and Health Laboratory, Faculty of Medicine and Pharmacy, Cadi Ayyad University, Marrakesh, MAR; 7 Department of Hematology and Bone Marrow Transplantation, Mohammed VI University Hospital, Marrakesh, MAR

**Keywords:** university hospital, medical departments, generic drugs, prescription, prevalence

## Abstract

A generic medication is a copy of an original drug for which the patent has expired. It contains the same active substances and is equivalent in terms of safety, efficacy, and pharmaceutical quality. Generic drugs are produced after the expiration of the brand-name drug's patent, which enables greater competition and reduces costs for patients and healthcare systems. They are subjected to strict regulatory and quality control standards to ensure compliance with pharmaceutical norms. This study aims to determine the current status of generic drug prescribing within the medical departments of the Mohammed VI University Hospital (UH) of Marrakesh.

This is a cross-sectional study with descriptive and analytical aims, involving 224 prescriptions issued in the medical departments of the Mohammed VI University Hospital (UH) of Marrakesh. To obtain the data required for the study, we included medical records, prescription sheets, and prescriptions delivered to hospitalized patients. In our study, 224 prescriptions were analyzed, with an overall total of 989 prescribed drugs, and a mean of 4.42 +- 2.39 drugs per prescription. Prescriptions from the Psychiatry Department accounted for 258 (26.09%) of total prescriptions, followed by those from the Cardiology Department at 130 (13.14%) and the Internal Medicine Department at 114 (11.53%). The generic prescribing rate for the UH's medical departments was 403 (40.75%). The Oncology Department had the highest generic prescribing rate (27 (64.29%)), followed by the Infectious Diseases and Rheumatology departments, at 29 (63.04%) and 34 (60.71%), respectively. In contrast, the Psychiatry Department had a generic prescribing rate of just 54 (20.93%). The most frequently prescribed classes as generic drugs were gastric antisecretory agents at 39 (100%), antiemetics at 32 (94.12%), and antivirals at nine (81.82%). The vast majority of drugs, 896 (90.59%), were reimbursable. In conclusion, we have noted that the generic drug prescription rate at the UH remains average compared with other institutions, and needs to be improved to optimize resources and control healthcare costs.

## Introduction

 A generic medication is a copy of an original drug for which the commercial exclusivity patent held by a pharmaceutical company has expired and that has become available in the public domain. Therefore, it is supposed to have the same pharmaceutical form and the same qualitative and quantitative composition of active substances as the reference product. This bioequivalence must be demonstrated by appropriate bioavailability studies [1].

The substitution of an originator molecule for its generic equivalent is of major interest because of the latter's low pricing, which encourages price competition and reduces costs in healthcare systems. The cost of generic drugs is generally low, as the preclinical and clinical studies required to develop an original drug are not necessary to demonstrate its safety and efficacy [2]. In addition, several generic drugs are often approved for the same product, which creates market competition and usually leads to lower prices [2,3]. In the United States, generic drugs have saved the healthcare system $2,200 billion in the period from 2009 to 2019 [2,4,5].

In terms of figures, the consumption of generic drugs in Morocco increased from 2.2 to 4 billion Dirhams between 2009 and 2018, marking an increase of nearly 82% [6]. In 2022, 70% of medicines sold on the Moroccan pharmaceutical market were generics, according to official statistics [7].

The subject of our work focuses on the share of generic drugs in hospital drug prescribing. Our main objective is to study the frequency of generic drug prescribing in the medical departments of the Mohammed VI University Hospital (UH) of Marrakesh, to establish an inventory of the situation. This article has already been presented as an oral abstract at the 22nd edition of the Residency Scientific Days in Marrakesh, on February 29, 2024.

## Materials and methods

Study type and duration

This is a cross-sectional study with descriptive and analytical aims, which took place over six months (from November 2021 to May 2022) and involved 224 prescriptions issued within the medical departments of the Mohammed VI UH of Marrakesh. The medical departments in question were Cardiology, Pneumology, Nephrology, Rheumatology, Neurology, Gastrology, Endocrinology and Metabolic Diseases, Dermatology, Internal Medicine, Infectious Diseases, Medical Oncology, Clinical Hematology, and Psychiatry.

Inclusion and exclusion criteria

All prescriptions issued within the medical departments of the Mohammed VI UH of Marrakesh and which contained at least one drug were collected, while those that did not originate from the medical departments of the UH of Marrakesh, contained only medical devices or included only hygienic-dietary rules were excluded.

Data collection

Data was collected from hospitalized patients' medical records, their prescription sheets, and prescriptions directly delivered to them, after obtaining authorization from the heads of departments and in collaboration with the residents in charge of the patients. We used a data collection form with two sections. The first section collected socio-demographic data, including age, sex, academic level, marital status, geographical origin, profession, and type of medical coverage. The second one covered data relating to drug prescriptions, such as drug name, galenic form, and administration route. For each drug, we specified the International Nonproprietary Name (INN) and therapeutic class using drug databases. We then determined the type of drug, generic or originator, and its reimbursement status using the CNOPS (Caisse Nationale des Organismes de Prévoyance Sociale/National Fund for Social Welfare Organizations) website.

Data entry and analysis

We used Microsoft Excel 2016 and SPSS software version 21.0 (IBM Corp., Armonk, NY) to create the database, process the data, and create the graphs. Descriptive analysis was performed to calculate means, standard deviations, and percentages. A comparison between qualitative variables was measured by Fisher's exact test. A p-value < 0.05 was considered significant for the study of the association between variables.

## Results

Socio-demographic data

A total of 224 prescriptions were included in the study, accounting for 989 prescribed drugs. The socio-demographic characteristics are summarized in Table 1. The mean age of patients at the time of the survey was 44.39 ± 16.47 years, with extremes of three and 87 years. The most represented age group was between 31 and 40 at 52 (23.21%), followed by the 41-50 group, which accounted for 48 (21.43%).

**Table 1:** Socio-demographic data

Characteristics		Number	Percentage (%)
Age mean (Years): 44.39± 16.47			
Age groups (Years)	0-10	2	0.89
	11-20	17	7.59
	21-30	25	11.16
	31-40	52	23.21
	41-50	48	21.43
	51-60	40	17.86
	61-70	28	12.50
	71-80	8	3.57
	81-90	4	1.78
Sex	Men	132	58.93
	Women	92	41.07
Origin	Rural	72	32.14
	Urban	152	67.86
Region	Marrakesh-Safi	187	83.49
	Draa-Tafilalet	13	5.80
	Beni Mellal- Khenifra	8	3.57
	Others	16	7.14
Profession	No	170	75.89
	Yes	54	24.11
Nature of profession (for patients who have a profession)	Employee	21	38.89
	Farmer	15	27.78
	Shopkeeper	7	12.96
	Government employee	7	12.96
	Artisan	4	7.41
Education	Never attended school	91	40.63
	Primary	50	22.32
	Secondary	68	30.35
	University	15	6.70
Marital status	Single	88	39.29
	Married	114	50.89
	Divorced	11	4.91
	Widowed	11	4.91
Health coverage	No	76	33.93
	Yes	148	66.07

Men accounted for 132 (58.93%) of the patients, with a sex ratio (M/F) of 1.43. Patients of urban origin accounted for 152 (67.86%). The majority of patients, 187 (83.49%), lived in the Marrakesh-Safi region (Table 1). Most of the patients (170 (75.89%)) were not professionally active. Of the professionally active ones (n=54), 21 (38.89%) were employees, 15 (27.78%) were farmers, seven (12.96%) were shopkeepers, seven (12.96%) were government employees and four (7.41%) were artisans. Ninety-one patients (40.63%) had never attended school, while 15 (6.70%) had a university education. Most patients, 114 (50.89%), were married, and 148 (66.07%) had health coverage (Table 1). A third of patients, i.e. 79 (35.27%), were admitted to the Psychiatry Department.

Drug prescription

**Figure 1:**
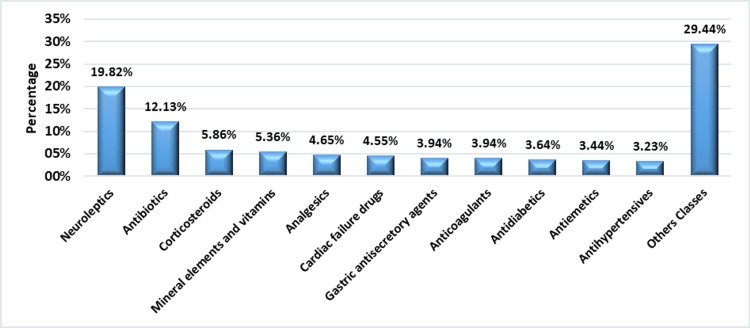
Distribution of medicines by therapeutic class The percentages are calculated using the total number of prescribed drugs (989) as the denominator.

A total of 989 drugs were prescribed, with a mean of 4.42 ± 2.39 drugs per prescription. Analysis revealed that the Psychiatry Department accounted for 258 (26.09%) of drug prescriptions, followed by the Cardiology Department with 130 (13.14%) and the Internal Medicine Department with 114 (11.53%). Neuroleptics were the most prescribed drug class, accounting for 196 (19.82%), followed by antibiotics at 120 (12.13%) and corticoids at 58 (5.86%) (Figure 1). In terms of reimbursement, 896 (90.60%) of drugs were reimbursable.

Generic drugs accounted for 403 (40.75%) of the 989 prescribed drugs, with 51 (22.78%) of the 224 prescriptions containing only originator drugs and 17 (7.59%) containing only generic drugs. The mean number of generic drugs per prescription was 1.80 ± 1.53. Sixty-three (28.13%) of prescriptions contained only one generic drug.

**Figure 2:**
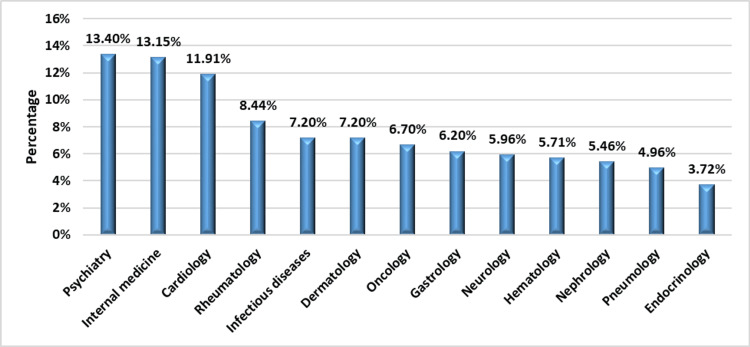
Distribution of generic prescriptions by department The percentages are calculated using the total number of generics (403) as the denominator.

The Psychiatry and Internal Medicine departments had the highest generic prescribing rates, with 54 (13.40%) and 53 (13.15%), respectively (Figure 2). The comparison between the rate of originator and generic drugs prescribed in each department is shown in Table 2. The classes most frequently prescribed as generics were gastric antisecretory agents at 39 (100%), anti-emetic drugs at 32 (94.12%), and antivirals at 9 (81.82%). These percentages were calculated by dividing the number of generic drugs in each class by the total number of drugs in that class, with the total number of gastric antisecretory agents being 39, anti-emetic drugs being 34, and antivirals being 11.

**Table 2:** Comparison of generic and originator prescribing rates by department The percentages are calculated using the total number in each department as the denominator.

Prescribing department	Total of prescribed drugs	Originators		Generics		P-value
		N	%	N	%	
Oncology	42	15	35.71	27	64.29	0.002
Infectious diseases	46	17	36.96	29	63.04	0.001
Rheumatology	56	22	39.29	34	60.71	0.002
Nephrology	43	21	48.84	22	51.16	0.157
Gastrology	50	25	50.00	25	50.00	0.185
Internal medicine	114	61	53.51	53	46.49	0.189
Endocrinology	33	18	54.55	15	45.45	0.59
Neurology	53	29	54.72	24	45.28	0.56
Dermatology	65	36	55.38	29	44.62	0.51
Hematology	52	29	55.77	23	44.23	0.66
Pneumology	47	27	57.45	20	42.55	0.87
Cardiology	130	82	63.08	48	36.92	0.38
Psychiatry	258	204	79.07	54	20.93	<0.001

The majority of generic drugs in our study were prescribed in tablet form (202 (50.12%)) and as injectable products (144 (35.73%)). Regarding the administration route of generic drugs, the oral route was the most common, accounting for 244 (60.55%), followed by the intravenous route at 134 (33.25%). Three hundred and fifty (86.85%) of the generic drugs were reimbursable.

## Discussion

In Morocco, generic drugs have been used since the 1970s, but their prescription remained restricted until recently. With the arrival of obligatory health insurance, the Ministry of Health has made considerable efforts to improve access to medicines for the population. Generics now hold an important place in the Moroccan pharmaceutical market [8]. Official statistics indicate that 70% of the drugs sold on the Moroccan pharmaceutical market are generics (3610 generic and bio-similar drugs out of a total of 5303) [7]. In addition, the pharmaceutical industries now devote a major part of their production to the manufacture and marketing of this category of products [9]. To the best of our knowledge, no study has examined the prescription and consumption of generics in Moroccan hospital settings.

In this study, the generic drug prescription rate in the medical departments of the UH of Marrakesh was evaluated at 40.75%. This result is in the mid-range compared with data from other studies. By comparing our results to other international studies, we noted that the generic prescribing rate at the Marrakesh UH is lower than that reported in some studies [10-12], and exceeds that observed in others [13,14]. Results vary considerably from one study to another, which highlights the importance of considering regional and contextual specificities when evaluating and promoting the use of generic drugs worldwide.

Prescribing rates for generic drugs are highly variable from one department to another. In Psychiatry, generic drugs are prescribed at a relatively low frequency, which could indicate a preference for originator drugs for reasons specific to this field of medicine, our findings are consistent with those in the literature [15,16]. Departments such as Pulmonology, Hematology, Dermatology, and Neurology show similar generic prescribing rates of approximately 45%, suggesting a certain preference for these more economical and equally effective alternatives. Other studies show a higher prevalence of generic prescribing in the dermatology department [17].

Generic drugs are often the source of negative prejudice and mistrust among healthcare professionals and patients alike. This can be explained by several factors, including poor knowledge of these drugs or bad experiences on the part of patients [18]. These prejudices can also be attributed to doubts about the quality of generic products.

Although generic drugs have demonstrated their equivalence to their originator equivalents, there are still doubts about the quality of these drugs, as the conditions for applying for marketing authorization have been eased for generics, for which pharmaco-toxicological and clinical files have been replaced by a bibliographical review. The problem arises for drugs with a narrow therapeutic index [19], where small variations in dose or concentration present a potential risk of compromising efficacy or safety. Thus, to ensure safe and effective use, it is necessary to adapt the dosage and monitor the patient [20]. 

The quality of medicines, whether originator or generic, is a key issue. Whilst many generic manufacturers are capable of producing good quality medicines, it is the responsibility of each country to guarantee the quality of medicines authorized to be marketed on its territory. Procedures and regulations ensure that generic drugs marketed in Morocco meet the same manufacturing standards as brand-name drugs. They are manufactured in pharmaceutical laboratories certified by the authorities and are subjected to the same regulations and manufacturing controls as brand-name drugs [6]. According to law 17/4, article 8, marketing authorization can only be granted if the drug has first passed appropriate tests designed to demonstrate its efficacy, guarantee its safety in normal conditions of use, and demonstrate its therapeutic benefit by establishing its bioequivalence to the reference drug. In addition, the manufacturer must provide proof of having carried out a qualitative and quantitative analysis of the drug, as well as possessing manufacturing methods and control procedures that guarantee the quality of the product during industrial manufacture.

Since the reference product sold in different countries may not be bioequivalent, each national market has its own regulations that determine which reference product should be used when developing and approving generic drugs [21]. For around 30 years, Morocco has had a quality assurance system in place that complies with Good Manufacturing Practices and current regulations. Under the new Pharmaceutical Code, drug manufacturers wishing to market generic drugs are authorized to carry out tests or experiments on the original pharmaceutical product before the patent protecting it expires [8].

Given the current state of medical and pharmaceutical legislation, the only boost to the development of generics in Morocco is their prescription by medical practitioners. The confidence of medical practitioners, therefore, needs to be won through scientific proof of the quality of generics, and this proof needs to be made available to them, along with up-to-date lists of equivalent generics. It is also a necessity, and even a duty, for public authorities to help doctors prescribe generics, as many of them cannot find their way around this panoply of generic products. Genuine promotion of generics will require a real policy in this direction, and Morocco can follow the example of developed countries that have succeeded with various policies in this area. This is a public health need in Morocco, as generic medicines are today the best solution for increasing access to medication.

In our study, the Oncology department recorded the highest rate of generic prescribing (64.29%), perhaps indicating the importance attached to cost and accessibility in a field where treatments can be particularly costly. A similar prevalence to ours has been reported in India; a study of anticancer prescribing showed a prevalence of 76.70% [22]. The rate of generic prescriptions was also high in the Infectious Diseases department (60.71%). 

Generic drug marketing offers multiple advantages, particularly in developing countries where originator drugs are often excessively expensive, with prices 50% to 80% higher than those of generics, making them unaffordable for many patients [23]. Thus, increasing the use of generic drugs generates significant savings within the Ministry of Health's budget. As per law 65-00, a reimbursement rate of 70% of the public price of the drug is applied based on its generic version, resulting in substantial savings [24]. The cost of generic drugs is systematically lower than that of originator drugs, and each time a new generic is launched on the market, its price falls even further, bringing significant benefits to patients [25]. Moreover, thanks to the savings they generate, generics play a crucial role in securing funding for research and therapeutic innovation [26,27].

These variations in generic prescribing rates between departments highlight the impact of factors such as medical preferences, institutional policies, and generic availability. Further analysis would be required to better understand the specific reasons behind these differences and to optimize the use of generics in each area.

Based on this study, several research topics emerge to deepen our understanding. First, an in-depth assessment of clinicians' knowledge of generic drugs could provide crucial information on the factors influencing generic prescribing. Secondly, an extended exploration of the correlation between generic prescribing and patients' socioeconomic status could provide data on the economic and social implications of this practice. These potential research topics would provide a better understanding of the aspects of generic prescribing and could contribute to strategic improvements in the promotion and use of these drugs.

Limitations and strengths of the study

This study has some worth mentioning limitations. Firstly, as a cross-sectional study, it provides a snapshot of the prevalence of generic prescribing but does not allow us to track changes in this prevalence over time. Secondly, excluding the surgical departments and those of the Mother and Child Hospital, as well as prescriptions during outpatient consultations and in day hospitals makes it difficult to obtain a complete picture of the prevalence of generic prescribing throughout the hospital. Finally, the small number of patients in each department probably made it difficult to obtain a statistically significant difference between generic and originator prescribing rates.

However, there are also some noteworthy strengths. The detailed analysis of generic drug prescribing at the Mohammed VI University Hospital in Marrakesh provides data directly applicable to this institution. The size of the sample (989 prescribed drugs) makes it robust and guarantees the representativeness and reliability of the results in the medical departments. Including various data sources, such as medical records, prescription forms, and inpatient prescriptions, ensures that the data is collected comprehensively and accurately. By covering various medical departments, the study provides an overview of variations and trends specific to each specialty. Identifying the generic prescribing rates for each department and the drug classes most often prescribed as generics provides valuable information for targeting optimization efforts to increase the generic prescribing rate. This helps to rationalize resources and control healthcare costs, offering direct added value for decision-makers and practitioners.

## Conclusions

In Morocco, the market for generic drugs has been growing steadily, as shown by the economic figures. This study, carried out in the medical departments of the UH of Marrakesh, sheds light on the practice of prescribing generic drugs in this healthcare institution. Our results revealed several key aspects of this practice that deserve to be highlighted. Firstly, it is clear that generic drug prescribing has become common practice at the UH of Marrakesh, but it should be noted that the prescribing rate remains average compared with other institutions, and needs to be improved in order to optimize resources and control healthcare costs. However, significant variations were observed in generic prescribing rates between different medical departments, underlining the importance of raising awareness and educating healthcare professionals in the departments with the lowest generic prescribing rates, such as the Psychiatry Department, on the benefits and equivalence of generic to brand-name drugs. Finally, it is essential to note that the promotion of generic drug prescribing is part of an overall approach aimed at improving the efficiency and accessibility of healthcare. As a leading healthcare facility, the UH of Marrakesh has the opportunity to play a major role in this initiative, by ensuring that generic prescribing is encouraged in a responsible and informed manner, with the ultimate aim of improving the quality of healthcare for the entire population served by this hospital establishment.

## References

[1] Biraben A, De Toffol B, Semah F, Rouaud T (2007). Use of generic anti-epilepsy drugs in France: survey of neurologists and review of the literature (Article in French). Rev Neurol (Paris).

[2] (2024). Generic Drug Facts. https://www.fda.gov/drugs/generic-drugs/generic-drug-facts.

[3] Conrad R, Lutter R (2024). Generic Competition and Drug Prices: New Evidence Linking Greater Generic Competition and Lower Generic Drug Prices. https://www.fda.gov/media/133509/download.

[4] (2024). Report Generic Drug and Biosimilars Access and Savings in the U.S.. https://accessiblemeds.org/2020-Access-Savings-Report.

[5] Conrad R, Kristin D (2024). Estimating Cost Savings from New Generic Drug Approvals in 2021. Estimating Cost Savings from New Generic Drug Approvals in.

[6] Conseil de la Concurrence du Maroc (2020). Avis du Conseil de la Concurrence relatif à la situation de la concurrence dans le marché du médicament au Maroc. https://conseil-concurrence.ma/wp-content/uploads/2023/10/Avis-du-Conseil-de-la-Concurrence-Num-A.4.20-FR-du-15-01-21-Web_compressed.pdf.

[7] Badrane M (2024). Medicines: Generics still suffering in the face of originator drugs. https://aujourdhui.ma/societe/medicaments-les-generiques-toujours-souffrants-face-aux-princeps.

[8] (2024). Politique Pharmaceutique Nationale. Médicaments et de la Pharmacie: Politique Pharmaceutique Nationale.

[9] Lemaizi S (2024). Médicament générique Le Maroc rate son objectif. https://www.cfcim.org/wp-content/uploads/2015/06/medicament.pdf.

[10] Ogbonna BO, Offor CA, Mgbemena BC (2020). Prevalence of generic drug prescribing in two tertiary health care facilities in south east Nigeria. Int J Pharm Pharmaceutical Res.

[11] Patel MS, Day SC, Halpern SD, Hanson CW, Martinez JR, Honeywell S Jr, Volpp KG (2016). Generic medication prescription rates after health system-wide redesign of default options within the electronic health record. JAMA Intern Med.

[12] Mambile G, Konje E, Kidenya BR, Katabalo D, Marwa K (2016). Quality of drug prescription in primary health care facilities in Mwanza, north-western Tanzania. Tanzan J Health Res.

[13] Wong JQ, Baclay JRM, Duque RG, Roque PMS, Serrano GKT, Tumlos JO, Ronsing AA (2014). The prevalence of Philippine prescribing, dispensing, and use behavior in relation to generic drugs and their risk factors. PIDS Discussion Paper Series.

[14] Mittal N, Mittal R, Singh I, Shafiq N, Malhotra S (2014). Drug utilisation study in a tertiary care center: recommendations for improving hospital drug dispensing policies. Indian J Pharm Sci.

[15] Chawla S, Agarwal M, Sharma S, Jiloha RC (2018). Drug utilization study of psychotropic drugs among psychiatric outpatients in a tertiary care hospital. Indian J Pharm Sci.

[16] Naliganti C, Valupadas C, Akkinepally RR, Eesam S (2019). Evaluation of drug utilization in cardiovascular disease at a teaching and referral hospital in Northern Telangana. Indian J Pharmacol.

[17] Lakshmi G, Priyanka C, Vineela M (2018). Drug utilisation pattern in dermatology outpatient department at a tertiary care hospital. Int J Basic Clin Pharmacol.

[18] Zaoui S, Hakkou F, Filali H, Khabal Y, Tazi I, Mahmal L (2013). Generic drug in Morocco: the point of view of the consumer (Article in French). Pan Afr Med J.

[19] (2024). Répertoire des médicaments génériques. https://ansm.sante.fr/documents/reference/repertoire-des-medicaments-generiques.

[20] Le Corre P (2010). Bioequivalence and generics of index drugs with narrow therapeutic margins (Article in French). Presse Med.

[21] Davit BM, Nwakama PE, Buehler GJ (2009). Comparing generic and innovator drugs: a review of 12 years of bioequivalence data from the United States Food and Drug Administration. Ann Pharmacother.

[22] Bepari A, Sakre N, Rahman I, Niazi SK, Dervesh AM (2019). The assessment of drug utilization study of anticancer drugs using WHO prescribing indicators in a Government Tertiary Care Hospital of the Hyderabad - Karnataka region of India. Open Access Maced J Med Sci.

[23] Randrianirina AF (2024). La perception des médicaments génériques par les utilisateurs : enquête pratique. https://toubkal.imist.ma/bitstream/handle/123456789/19173/P0942021.pdf?sequence=1.

[24] Agence national de l’assurance maladie. Maroc (2024). Agence national de l’assurance maladie. https://www.acaps.ma/sites/default/files/loi-65-00.pdf.

[25] (2024). Décret n° 2-13-852 du 14 safar 1435 (18 décembre 2013) relatif aux conditions et aux modalités de fixation du prix public de vente des médicaments fabriqués localement ou importés. https://www.sante.gov.ma/Reglementation/TARIFICATION/2-13-852.pdf.

[26] (2024). Enjeux autour du médicament générique. Ministère Trav. Santé Solidar. https://sante.gouv.fr/soins-et-maladies/medicaments/professionnels-de-sante/medicaments-generiques-a-l-usage-des-professionnels/article/enjeux-autour-du-medicament-generique..

[27] Quedinel M (2024). Les médicaments génériques, et maintenant les médicaments hybrides, une chance pour pérenniser le système de santé français. https://dumas.ccsd.cnrs.fr/dumas-03205350v1/file/Ma%C3%BFlis%20QUEDINEL.pdf.

